# The combined effect of fish oil containing Omega‐3 fatty acids and *Lactobacillus plantarum* on colorectal cancer

**DOI:** 10.1002/fsn3.3037

**Published:** 2022-09-28

**Authors:** Elahe Sharifi, Zahra Yazdani, Mojtaba Najafi, Zahra Hosseini‐khah, Ali Jafarpour, Alireza Rafiei

**Affiliations:** ^1^ Department of Fisheries, Faculty of Marine Sciences Chabahar Maritime University Chabahar Iran; ^2^ Department of Immunology, Molecular and Cell Biology Research Center, School of Medicine Mazandaran University of Medical Sciences Sari Iran; ^3^ Student Research Committee Mazandaran University of Medical Sciences Sari Iran; ^4^ Genetics and Animal Breeding Department Gorgan University of Agricultural Sciences and Natural Resources Golestan Iran; ^5^ Diabetes Research Center Mazandaran University of Medical Sciences Sari Iran; ^6^ Department of Fisheries, Faculty of Animal Science and Fisheries Sari Agricultural Sciences and Natural Resources University Sari Iran; ^7^ Food R&D team UPSIDE FOODS Inc Berkeley USA

**Keywords:** co‐encapsulation, colorectal cancer, *Lactobacillus plantarum*, omega‐3 fatty acids

## Abstract

Colorectal cancer (CRC) is one of the deadliest malignancies. Recent attempts have indicated the role of diet in the etiology of CRC. Natural dietary compounds such as probiotics and Omega‐3 fatty acids that act synergistically can be beneficial in finding a tremendous solution against CRC. To date, the combined effect of fish oil containing Omega‐3 fatty acids (Omega‐3) and *Lactobacillus plantarum* (*L. plantarum*) on CRC has been left behind. We here evaluated the effects of co‐encapsulation of Omega‐3 and probiotic bacteria on CRC cell lines compared to normal cells. Omega‐3 and *L. plantarum* bacteria were co‐encapsulated in three ways, including gelatin–gum Arabic, gelatin–chitosan, and chitosan–gum Arabic complex coacervate microcapsules. After treatment of cells (Normal [L929] and colorectal [C26]) by *L. plantarum,* Omega‐3, and microcapsules, viability and growth capacity of cell lines were measured using the MTT (3‐[4, 5‐dimethylthiazol‐2‐yl]‐2, 5‐diphenyltetrazolium bromide) assay. Isolated total RNA was used to evaluate the expression profile of BCL2‐associated X protein (BAX), B‐cell lymphoma 2 (BCL‐2), and Caspase‐3 (CASP3) genes by real‐time polymerase chain reaction (PCR). Statistical analysis was performed with SPSS 25 software. A value of *p* < .05 was considered statistically significant. The results indicated a significant reduction in cell viability of C26 in a concentration‐dependent manner in the treated cells with all treatments, except gelatin–gum Arabic microcapsules. The messenger RNA (mRNA) expression level of the BAX and CASP3 genes in C26 cells being treated with all treatments significantly increased than in untreated cells, and the expression level of the anti‐apoptotic factor of the BCL‐2 gene decreased in C26 cells simultaneously (*p* < .05). Although, the combined effect of Omega‐3 and *L. plantarum* and microcapsulated treatments had no more effect on viability and apoptosis gene expression of cancer cells compared to Omega‐3 or *L. plantarum*. In conclusion, combination therapy with fish oil containing Omega‐3 and *L. plantarum* does not improve the anticancer effect of each alone.

## Graphical abstract

The present study showed that Omega‐3 selectively induces cytotoxicity and apoptosis in tumor cells. In addition, the suspension of L. Plantarum and bacteria extract induced apoptosis in cancer cells more than normal cells. The combined effect of L. Plantarum and Omega‐3 did not improve cytotoxicity and apoptosis in colorectal C26 cancer cells compared with monotherapy with L. Plantarum or Omega‐3.

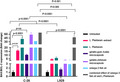

## 1 INTRODUCTION

Colorectal cancer (CRC) is the third most commonly diagnosed cancer type and the second most common cause of cancer death worldwide, with almost 2 million new cases in 2020 years (Sung et al., 2021). Environmental factors like particular dietary, lifestyle, and genetic factors can facilitate polyp growth and the development of CRC by promoting intestinal inflammation and changing the microbiome of the colorectal (Rafter & Gastroenterology, 2003). Due to the complex relationship between microflora and CRC development, finding the mechanisms by which the bacterial community impacts CRC could introduce a new therapeutic approach. However, detecting a particular bacterial community or even modifying the abundance of one strain responsible for CRC development is not easy (Rafter & Gastroenterology, 2003). Recent studies have revealed that probiotic consumption affects the diversity and richness of microbiota and can reduce chronic inflammation (Liu et al., 2011; Ohara et al., 2010). Among the identified probiotic bacteria strains, *Lactobacillus plantarum* (*L. plantarum*) is well documented based on its activity and anti‐inflammatory and anticarcinogenic role in cells (Dong et al., 2012; Evrard et al., 2011). It causes apoptosis by inducing reactive oxygen species (ROS), overexpression of pro‐apoptotic proteins of BAX, cytochrome C (Cyt C), Caspase‐3 (CASP3), Caspase‐8 (CASP8), and Caspase‐9 (CASP9), and inhibition of anti‐apoptotic protein of B‐cell lymphoma 2 (BCL‐2) (Kim et al., 2015; Sun et al., 2021). However, the clinical efficacy of probiotics remains low due to reducing their survival rate during processing, storage, and passage through the gastrointestinal (GI) tract (Razavi et al., 2021). Exposure to low gastric pH, oxygen, and temperature in the GI tract can influence quantities of probiotic bacteria at the site of action. Nowadays, microencapsulation of probiotics has been considered an appropriate way to improve the survival rate and protection of probiotics in the GI tract (Razavi et al., 2021). Microencapsulation is a process to protect cells from injury or loss by retaining cells within a specific encapsulating membrane (Krasaekoopt et al., 2003). On the other hand, Omega‐3 fatty acids (Omega‐3) in fish oil, such as eicosapentaenoic acid (EPA) and docosahexaenoic acid (DHA), have anticancer effects (Gutiérrez et al., 2019). They can decrease cell viability and induce pro‐apoptotic pathways in CRC (Cockbain et al., 2012; Volpato & Hull, 2018). Previous investigations have shown a synergetic effect between probiotic bacteria and Omega‐3 in the GI tract by enhancing the binding probiotic to the intestinal wall (Eratte et al., 2015). Additionally, a study showed that the viability of bacteria increased when they were co‐encapsulated with tuna oil compared to being encapsulated on their own (Eratte et al., 2015). To date, the combined effect of co‐encapsulated Omega‐3 and probiotic bacteria on CRC cell lines has not been reported. Therefore, this study aimed to evaluate the effect of Omega‐3 and *L. plantarum* combination therapy on survival and expression of apoptosis‐related genes in the C26 colorectal cancer (CRC) cell line compared to that in a normal L929 fibroblast cell line.

## 2 MATERIAL AND METHODS

### 2.1 Chemicals and reagents

*Lactobacillus plantarum*, a gram‐positive lactic acid bacterium, can mainly be found in fermented food and the GI tract and nowadays it is utilized in the food industry as a probiotic. *L. plantarum* bacteria (*PTCC 1058*) was purchased from the Biotechnology Department in the Iranian Research Organization for Science and Technology (IROST), Iran. Omega‐3 dietary supplement (fish oil) was purchased from Golden Seas company (Yazd, Iran). According to data provided by the supplier, the major fatty acids in this fish oil were as follows: 240 mg DHA and 360 mg EPA. Gelatin, chitosan, and gum Arabic, and other materials were purchased from Sigma‐Aldrich Ltd., Australia. MTT (3‐[4, 5‐dimethylthiazol‐2‐yl]‐2, 5‐diphenyltetrazolium bromide) and dimethyl sulfoxide (DMSO) were purchased from Sigma‐Aldrich, Germany. Dulbecco's modified Eagle's medium (DMEM) and Penicillin‐Streptomycin were obtained from Biowest, Germany. Fetal bovine serum (FBS) and trypsin were purchased from Gibco, Germany. Cell culture plates and flasks were purchased from SPL, Korea, and the microtubes were purchased from Ratiolab, Germany. All primers were designed and purchased from DNA Pioneer, Korea. RNA extraction kit and complementary DNA (cDNA) synthesis kit were purchased from FAVORGEN, Taiwan, and Addbio, Korea.

### 2.2 Preparation of *L. plantarum* culture and *L. plantarum* extract

The *L. plantarum* was cultured for 48 h in MRS (Man, Rogosa, and Sharpe**)** broth at 35°C. All the inoculation works were done under laminar air flow. Finally, the cells were harvested at the stationary growth phase by centrifuging at 1500 rpm for 15 min. The supernatant was separated and considered as the *L. plantarum* extract. After discarding the supernatant, the precipitated cell mass was washed twice with sterile Normal saline (0.9% NaCl) to obtain *L. plantarum* suspension.

### 2.3 Microencapsulation preparation

The complex coacervation and microencapsulation procedures were carried out as detailed in our previous study (In press). Briefly, Omega‐3 and *L. plantarum* were co‐encapsulated in three ways, including gelatin–gum Arabic, gelatin–chitosan, and chitosan–gum Arabic complex coacervate microcapsules with the wall to a core ratio of 4:1. Microcapsules were prepared with a high‐speed homogenizer at 7200 rpm for 10 min and an equal proportion of bacteria and Omega‐3 fish oil (1:1).

### 2.4 Cell culture and viability assay

Normal subcutaneous connective tissue (L929) and colorectal mouse (C26) cell lines were purchased from Pasteur Institute (Tehran, Iran) and cultured in DMEM low glucose media containing 10% FBS and antibiotic (containing 50 U/ml Penicillin and 50 μg/ml of Streptomycin). These cells have fibroblast morphology. The cells were incubated at 37°C in 90% humidity containing 5% CO_2_. After the cells reached 70% confluence, they were dissociated by trypsin 0.25% and centrifuged at 1800 rpm for 5 min (Yazdani et al., 2020). The MTT assay was used for measuring the viability of cells. First, 8 × 10^3^ cells/well of C26 and 1 × 10^4^ cells/well of L929 were cultured on 96‐well plates. After 24 h, cells were treated with *L. plantarum*, *L. plantarum* suspension, Omega‐3, gelatin–gum Arabic microcapsule, gelatin–chitosan microcapsule, chitosan–gum Arabic microcapsule, and combination therapy of Omega‐3 and *L. plantarum* (with concentrations of 25%, 50%, 75%, and 100% from stock treatments in Table 1). The untreated cells are considered a control group. After 24 h, the cells in each study group were treated with 20 μl of the MTT reagent (5 mg/ml in sterile phosphate‐buffered saline [PBS]) and incubated at 37°C for 4 h. Finally, the culture medium was removed, and 200 μl of DMSO was added to dissolve the formazan crystals. The results were measured at the absorbance of 570 nm in a microplate reader (Bio‐Tek Instruments, Inc., Vermont, USA). The percentage of cell viability was calculated following the formula:

**TABLE 1:** Co‐encapsulation efficiency of Omega‐3 and viability of *L. plantarum*

Microcapsule	Co‐encapsulation efficiency of Omega‐3 fatty acids (%)	Bacteria count before micro‐capsulation	Bacteria count* after micro‐capsulation
Gelatin–gum Arabic	85.58 ± 0.12	2.4 × 10^8^ cfu/ml	6.37 × 10^6^ ± 0.02^a^
Gelatin–chitosan	96.26 ± 0.19	2.4 × 10^8^ cfu/ml	7.50 × 10^3^ ± 0.19^b^
Gum Arabic–chitosan	90.70 ± 0.15	2.4 × 10^8^ cfu/ml	1.88 × 10^4^ ± 0.001^b^

* Values within a column with different superscripts differ *p* < .05.

% viability = absorbance test/absorbance control × 100 (Yazdani et al., 2020).

### 2.5 Gene expression

The L929 and C26 cell lines were cultured at 2 × 10^6^ cells per well in 6‐well plates. After 24 h, cells were treated with an IC50 concentration of *L. plantarum* bacteria, *L. plantarum* extract, Omega‐3, gelatin–gum Arabic microcapsule, gelatin–chitosan microcapsule, chitosan–gum Arabic microcapsule, and combination therapy of Omega‐3 and *L. plantarum*, and untreated cells considered as controls. Total RNA was extracted and cDNA was synthesized using Addbio cDNA Synthesis Kit, Korea, based on the manufacturer's protocols. The synthesized cDNA was directly used as a template for real‐time quantitative polymerase chain reaction (RT‐qPCR). The primer design was performed by Oligo 7.0 software for BAX, BCL2, and CASP3 genes and glyceraldehyde 3‐phosphate dehydogenase (GAPDH) as a reference housekeeping gene (Yazdani et al., 2021). It is noticed that only a specific reverse primer was used for cDNA synthesis. Therefore, for the TaqMan real‐time quantitative PCR (RT‐qPCR), forward primers were used and Bio‐Rad CFX qPCR Instrument was used to carry out a real‐time PCR. Amplification reactions were performed in a 15 μl reaction volume containing 13.5 μl Master Mix, 0.5 μl of specific forward primer (10 μM), 1 μl of cDNA, 2 μl of H_2_O. Each sample was tested in triplicate. The optimized PCR conditions were 95°C for 15 min, followed by 45 cycles of 95°C for 15 s, and 60°C for 1 min. The levels of mRNA expression were normalized to GAPDH and were calculated using the 2^−ΔΔC*t*^ method (Yazdani et al., 2021).

### 2.6 Statistical analysis

All data were presented as mean ± standard error. Statistical significance of differences between mean values was analyzed by one‐way analysis of variance (ANOVA) followed by Tukey's test using IBM SPSS Statistics 25 software. A value of *p* < .05 was considered statistically significant.

## 3 RESULTS

### 3.1 Co‐encapsulation efficiency

The complex coacervation and microencapsulation procedures were carried out as detailed in our previous study (In press). Briefly, the viability of *L. plantarum* in gelatin–gum Arabic, gelatin–chitosan, and chitosan–gum Arabic microcapsules is presented in Table 1.

The viability of *L. plantarum* was significantly higher in gelatin–gum Arabic microcapsules compared to that in other microcapsules. Also, the microencapsulation efficiency of Omega‐3 fatty acids showed that the biopolymer mixture containing gelatin–chitosan microcapsules has the highest yield (96.26 ± 0.19). Our results showed that using the combined coacervation technique with fish gelatin biopolymers, chitosan, and gum Arabic as wall materials can produce Omega‐3 capsules and *L. plantarum* in micrometer sizes following the protocol described in our previous study (In press).

### 3.2 Cytotoxicity test

**FIGURE 1:**
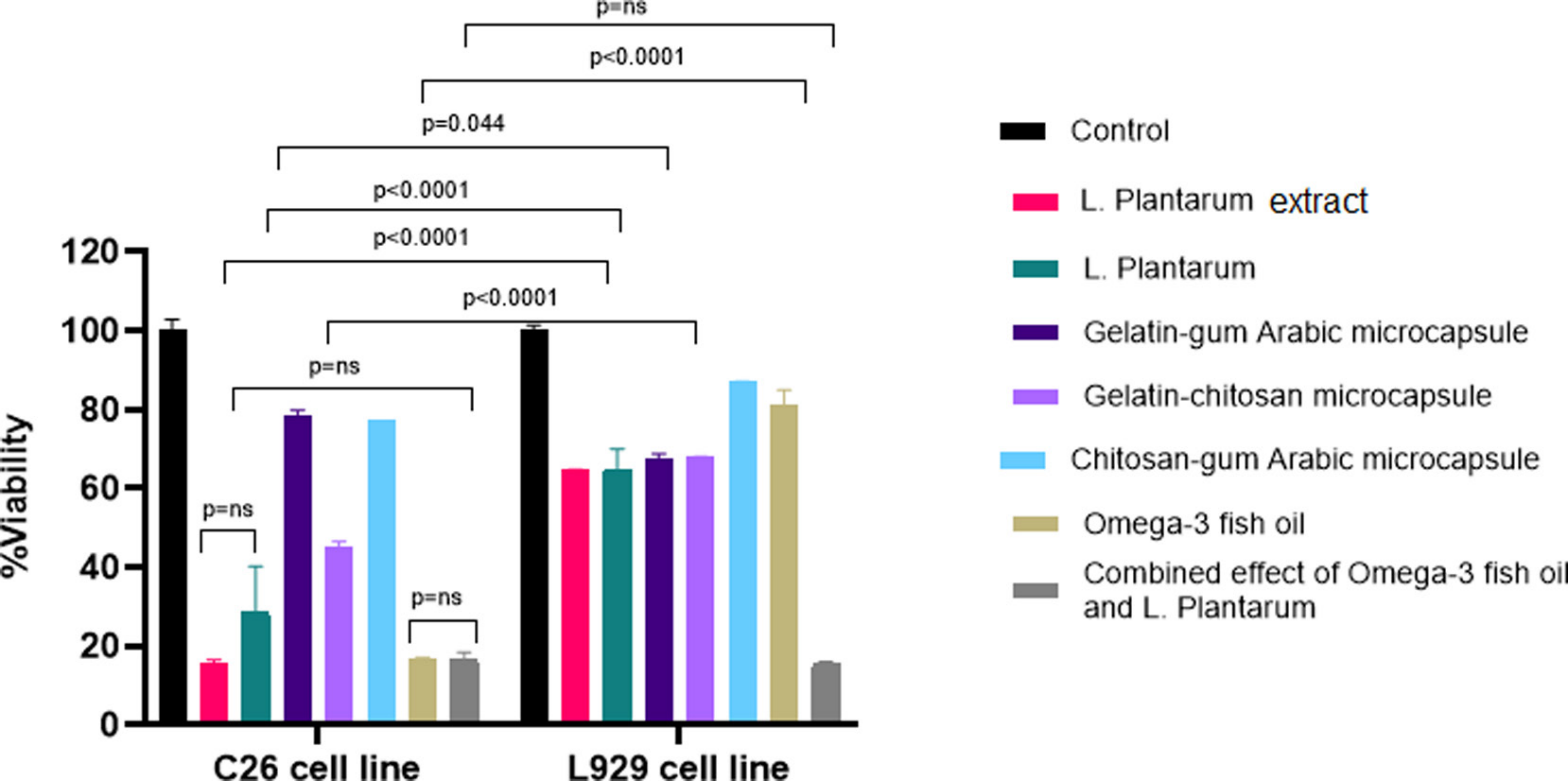
The effect of different treatments on cell viability in C26 and L929 cell lines using the (3‐[4, 5‐dimethylthiazol‐2‐yl]‐2, 5‐diphenyltetrazolium bromide) (MTT) assay. Both cell lines were treated with a suspension of *L. plantarum*, *L. plantarum*, gelatin–gum Arabic microcapsule, gelatin–chitosan microcapsule, chitosan–gum Arabic microcapsule, Omega‐3, and the combined effect of Omega‐3 and *L. plantarum*, respectively. (ns = Non significant)

The cytotoxic effect of different treatments on normal (L929) and cancer (C26) cells was tested using MTT assay. Figure 1 shows the viability of C26 cells treated with *L. plantarum*, *L. plantarum* extract, Omega‐3, gelatin–chitosan microcapsule, chitosan–gum Arabic microcapsule significantly decreased compared to L929 cells. No significant difference was observed for two *L. plantarum* and its extract treatments. The combined effect of Omega‐3 and *L. plantarum*, gelatin–gum Arabic microcapsule, gelatin–chitosan microcapsule, and chitosan–gum Arabic microcapsule had no more cytotoxicity on the C26 cell line in comparison with *L. plantarum* and Omega‐3 treatment.

**FIGURE 2:**
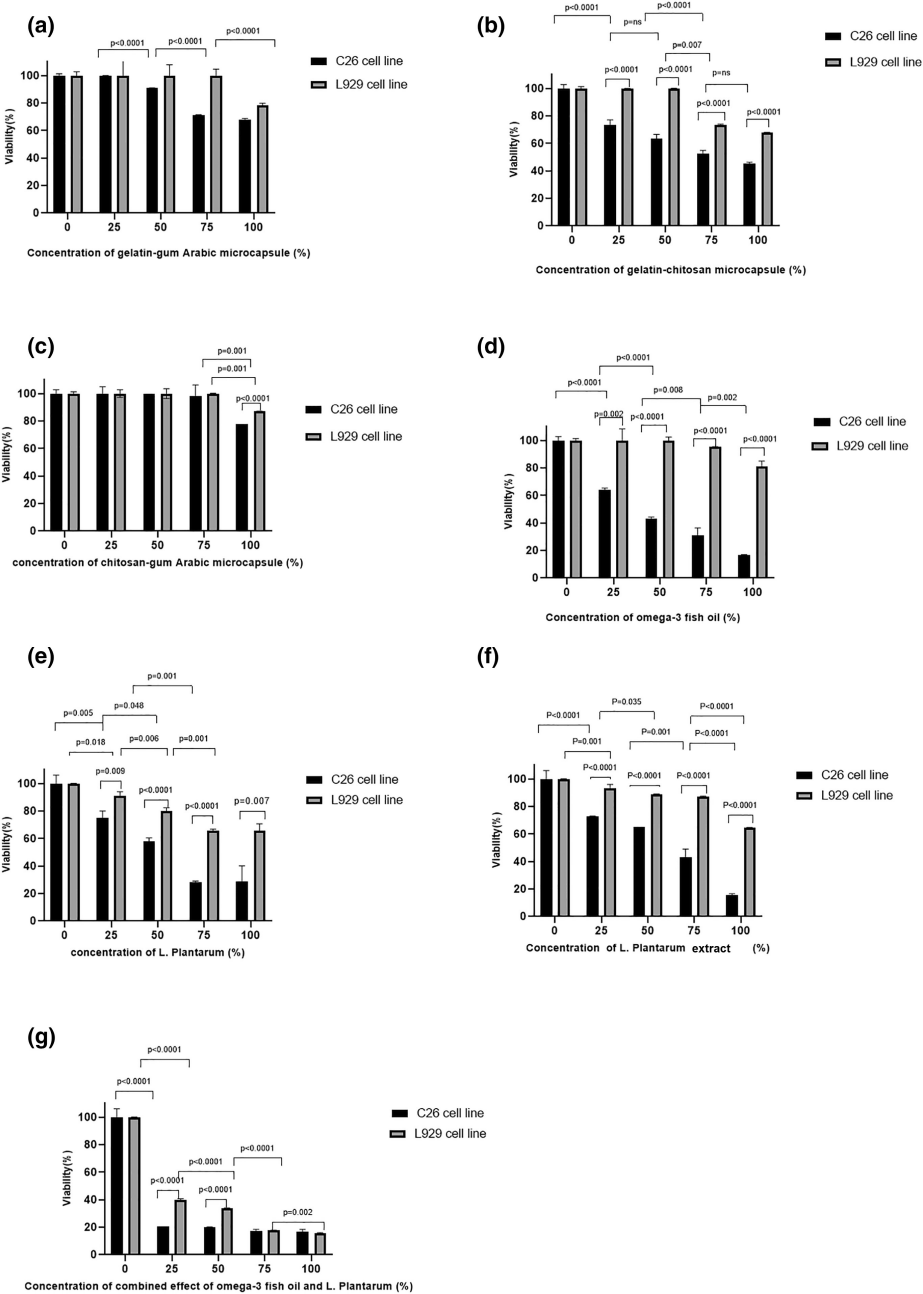
The effect of different concentrations of (a) gelatin–gum Arabic microcapsule, (b) gelatin–chitosan microcapsule, (c) chitosan–gum Arabic microcapsule, (d) Omega‐3, (e) *L. plantarum*, (f) suspension of *L. plantarum*, and (g) the combined effect of Omega‐3 and *L. plantarum* on the cell viability of C26 and L929 cell lines using the (3‐[4, 5‐dimethylthiazol‐2‐yl]‐2, 5‐diphenyltetrazolium bromide) (MTT) assay

Figure 2 shows the viability of cells that were treated with different concentrations of each treatment. The cells treated with Omega‐3, *L. plantarum*, and *L. plantarum* extract showed an inhibition effect on cell viability, especially in C26 cells in a concentration‐dependent manner. No difference was observed in cancer and normal cells treated with chitosan–gum Arabic microcapsule and it decreased the viability of cancer cells only in the highest concentration. In the samples treated with gelatin–chitosan microcapsules, the cell viability rate of C26 significantly decreased with the increase in concentration. The viability of L929 cells treated with gelatin–gum Arabic microcapsules was similar to the control, while there was an inhibitory effect on the cell viability of C26 cells in a concentration‐dependent manner. Moreover, the MTT assay results revealed a remarkable decrease in cell viability of C26 cells at all treatments, except gelatin–gum Arabic microcapsules. Table 2 shows the IC50 of each treatment on the C26 cell line.

**TABLE 2:** IC50 of each treatment of gelatin–gum Arabic microcapsule, gelatin–chitosan microcapsule, chitosan–gum Arabic microcapsule, Omega‐3 fish oil, *L. plantarum*, and *L. plantarum* extract on the C26 cancer cell line

Treatment	IC50 on C26 (mg/ml)
Gelatin–gum Arabic microcapsule	9.4 (mg/ml)
Gelatin–chitosan microcapsule	8.506 (mg/ml)
Chitosan–gum Arabic microcapsule	17.61 (mg/ml)
Omega‐3 fish oil	13.53 (mg/ml)
*L. plantarum*	0.5172 × 10^7^ (cfu/ml)
*L. plantarum* extract	0.8491 × 107 (cfu/ml)
Combination of Omega‐3 and *L. plantarum*	0.08491 × 10^7^(cfu/ml) 1.35 (mg/ml)

### 3.3 Expression of apoptosis‐related genes

**FIGURE 3:**
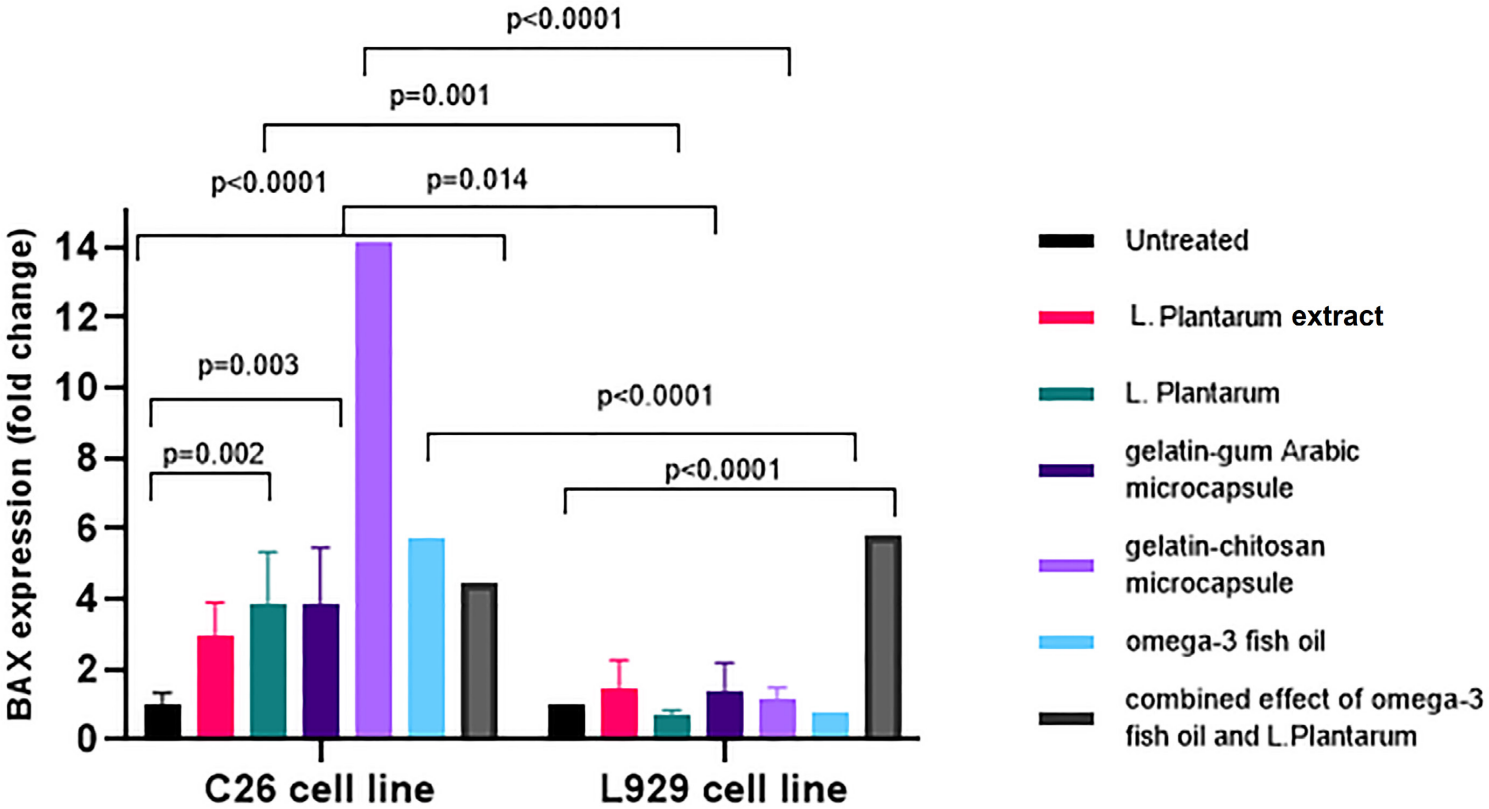
The effect of different treatments of *L. plantarum*, suspension of *L. plantarum*, gelatin–gum Arabic microcapsule, gelatin–chitosan microcapsule, Omega‐3, and the combined effect of Omega‐3 fish oil and *L. plantarum* on gene expression profiling of BCL2‐associated X protein (BAX) in C26 and L929 cells

A relative quantitative real‐time PCR method was applied to assess BAX, BCL‐2, and CASP3 gene expression levels in all treatments, which have a cytotoxic effect on the C26 cell line, including gelatin–gum Arabic microcapsule, gelatin–chitosan microcapsule, Omega‐3, *L. plantarum*, extract of *L. plantarum*, and the combined effect of Omega‐ 3 and *L. plantarum*. The results indicated that the mRNA level of the BAX gene was significantly increased in C26 cells treated with *L. plantarum*, Omega‐3, chitosan, gelatin–gum Arabic microcapsules, and gelatin–chitosan microcapsules rather than in L929 cells. Interestingly, the mRNA expression level of the BAX gene was increased at C26 treated with gelatin–chitosan microcapsules by 14.0 times compared to the L929 cells. Gelatin–gum Arabic microcapsules and the combined effect of Omega‐3 *and L. plantarum* had no more effect on gene expression of the C26 cell line than Omega‐3 or *L. plantarum*. Gelatin–gum Arabic microcapsule, gelatin–chitosan microcapsule, *L. plantarum*, and Omega‐3 increased BAX gene expression in C26 cells in comparison with the L929 cell line (Figure 3).

**FIGURE 4:**
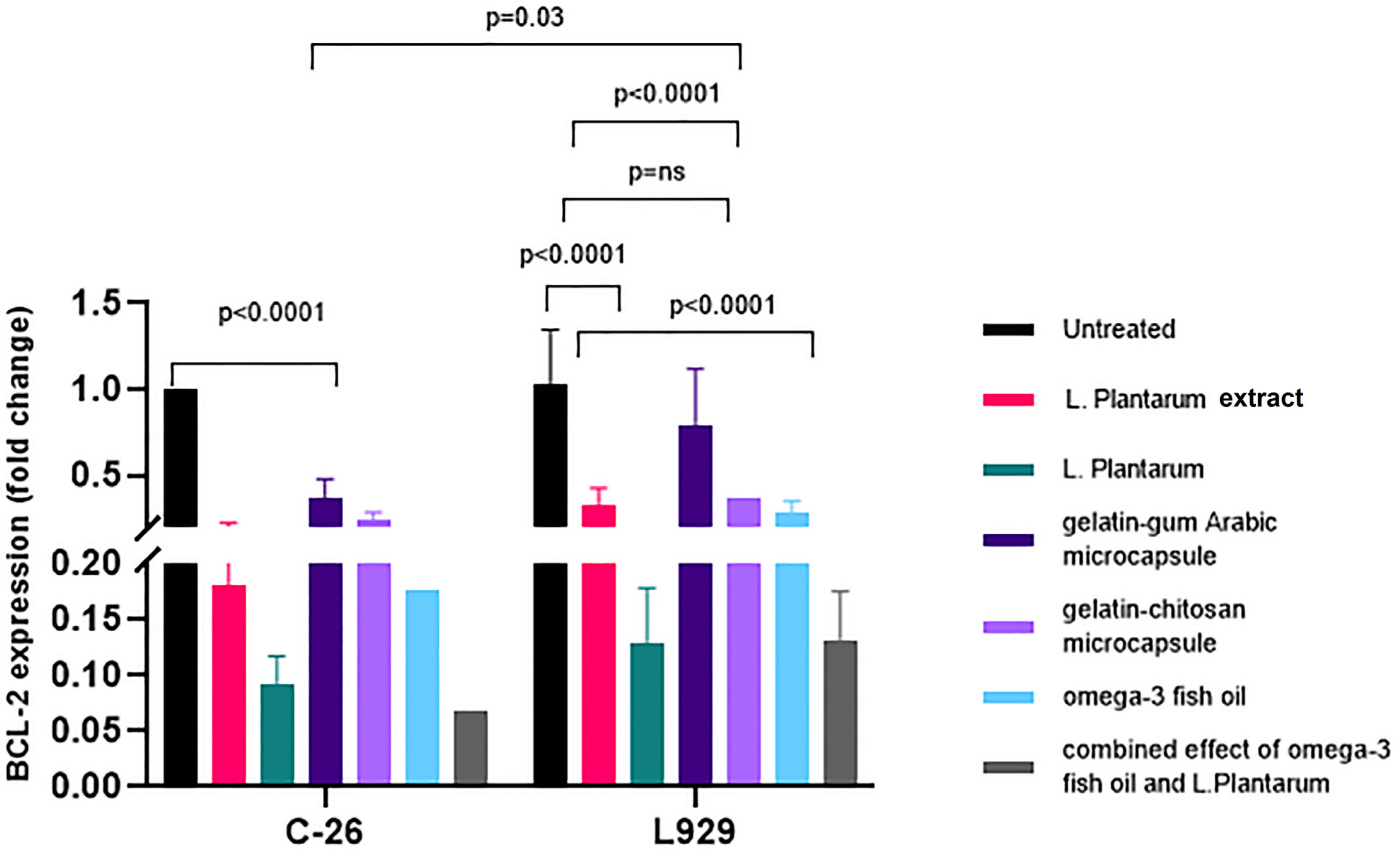
The effect of different treatments of *L. plantarum*, suspension of *L. plantarum*, gelatin–gum Arabic microcapsule, gelatin–chitosan microcapsule, Omega‐3, and the combined effect of Omega‐3 and *L. plantarum* on the gene expression profiling of B‐cell lymphoma 2 (BCL‐2) in C26 and L929 cells

The mRNA levels of the BCL‐2 gene were significantly decreased in C26 cells treated with *L. plantarum*, Omega‐3, chitosan, gelatin–gum Arabic microcapsules, gelatin–chitosan microcapsules rather than untreated cells. Gelatin–gum Arabic microcapsules and the combined effect of Omega‐3 and *L. plantarum* had no more effect on gene expression in comparison with Omega‐3 or *L. plantarum*. In the L929 cell line, only the combined effect of Omega‐3 and *L. plantarum* had more effect on gene expression of BAX in comparison with untreated cells. Gum Arabic microcapsule decreased the BCL‐2 gene expression in the C26 cells compared to the L929 cell line (Figure 4).

**FIGURE 5:**
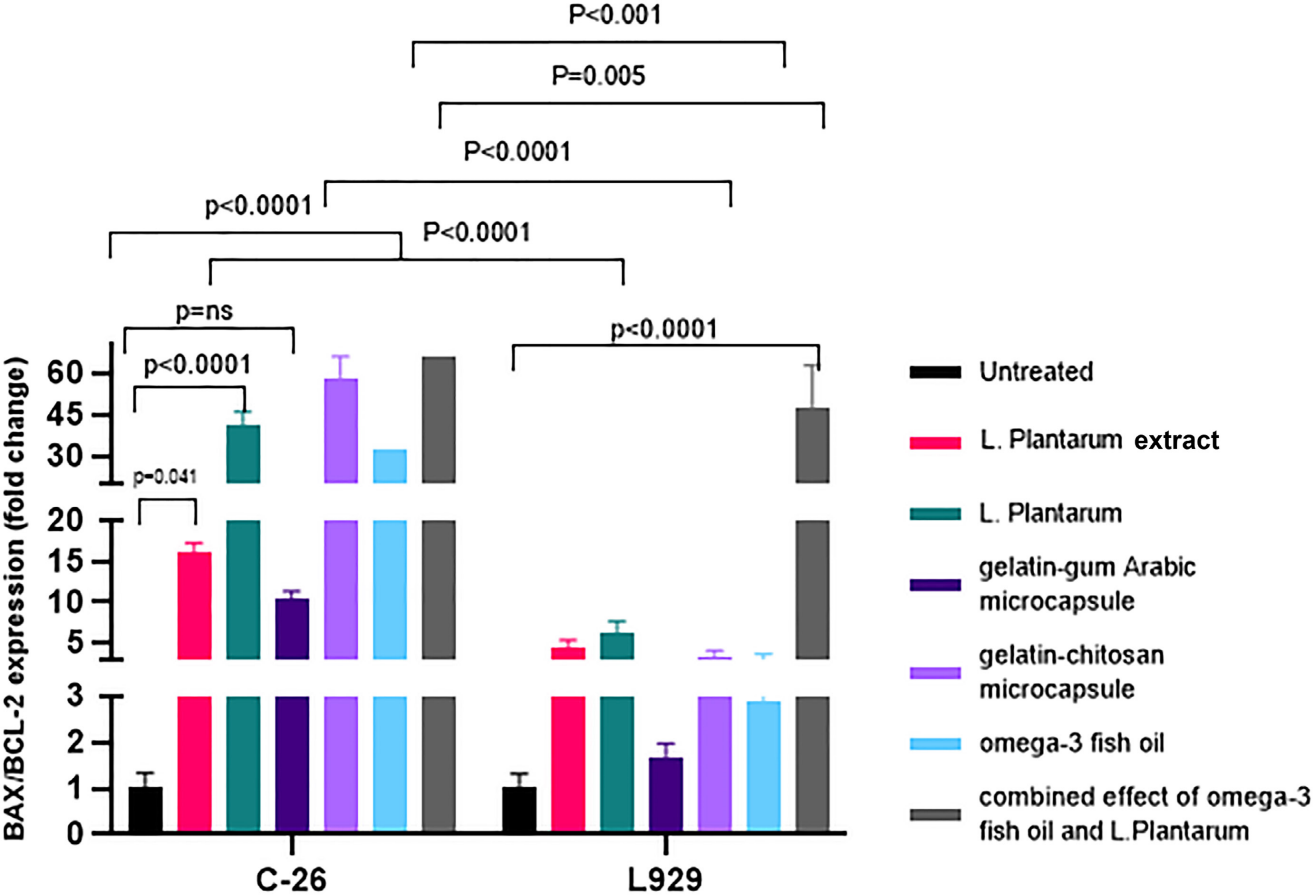
BCL2‐associated X protein/B‐cell lymphoma 2 (BAX/BCL‐2) relative expression in C26 and L929 cells treated with different treatments of *L. plantarum*, suspension of *L. plantarum*, gelatin–gum Arabic microcapsule, gelatin–chitosan microcapsule, Omega‐3, and the combined effect of Omega‐3 and *L. plantarum*

According to the obtained results, BAX/BCL‐2 mRNA ratio was significantly increased in the C26‐treated cells compared to untreated cells. Gelatin–chitosan microcapsule, gelatin–gum Arabic microcapsules, and the combined effect of Omega‐3 and *L. plantarum* was not more effective in gene expression of the C26 cell line in comparison with Omega‐3 or *L. plantarum* bacteria. *L. plantarum*, gelatin–gum Arabic microcapsule, gelatin–chitosan microcapsule, and Omega‐3, and the combined effect of Omega‐3 and *L. plantarum* increased the BAX/BCL‐2 ratio in C26 cells in comparison with the L929 cell line (Figure 5).

**FIGURE 6:**
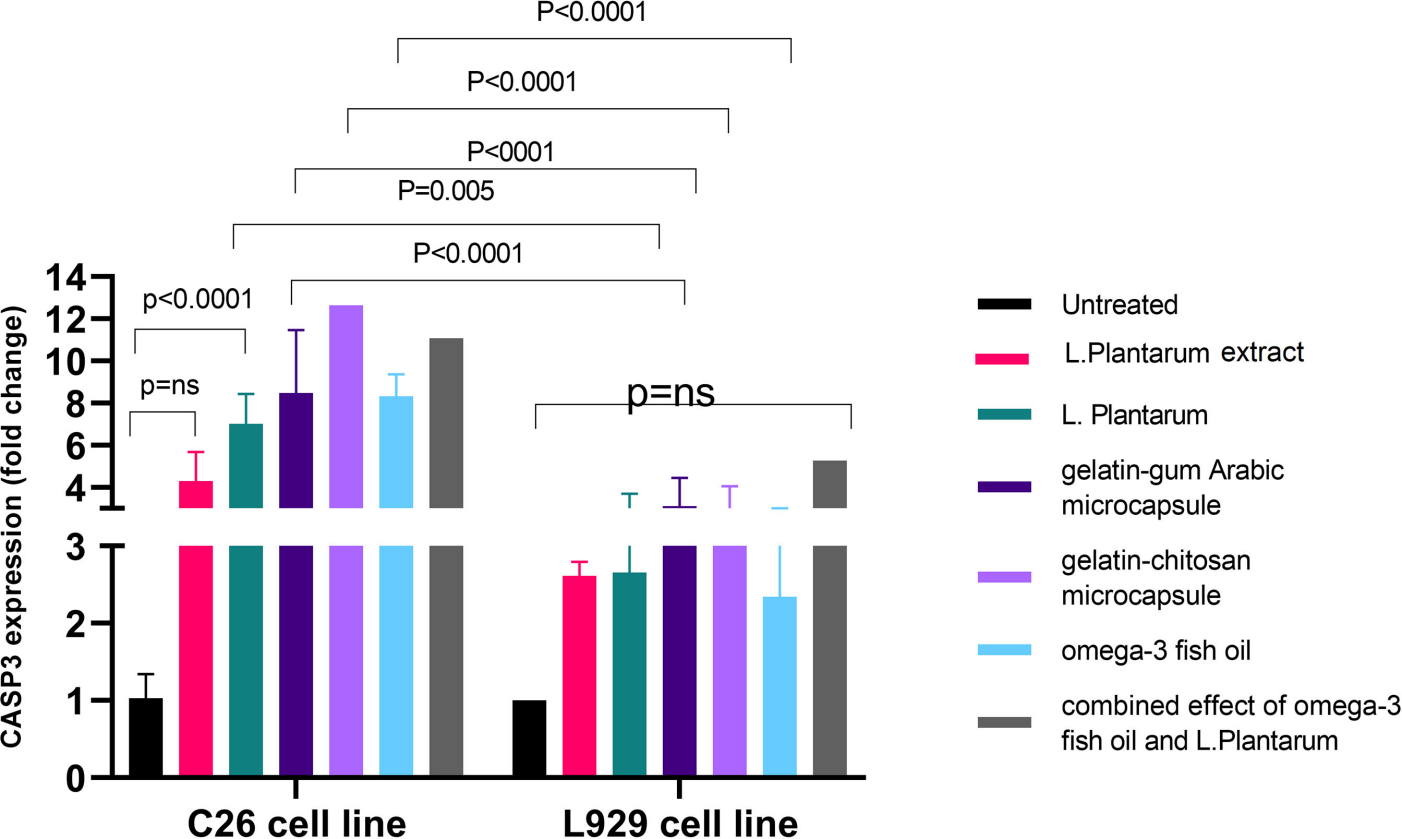
Caspase‐3 (CASP3) relative expression in C26 and L929 cells treated with different treatments of *L. plantarum*, suspension of *L. plantarum*, gelatin–gum Arabic microcapsule, gelatin–chitosan microcapsule, Omega‐3, and the combined effect of Omega‐3 and *L. plantarum*

Expression analysis revealed that CASP3 had higher gene expressions in C26‐treated cells than untreated cells in all groups. However, gelatin–gum Arabic microcapsules, gelatin–chitosan microcapsules, and the combined effect of Omega‐3 and *L. plantarum* had no more effect on gene expression in comparison with Omega‐3 or *L. plantarum*. Gelatin–gum Arabic microcapsule, gelatin–chitosan microcapsule, *L. plantarum*, Omega‐3, and the combined effect of Omega‐3 and *L. plantarum* increased the CASP3 gene expression in C26 cells in comparison with the L929 cell line (Figure 6).

## 4 DISCUSSION

In the present study, the findings manifested that Omega‐3 exhibited potent antitumor effects on C26 cancer cells. Previous studies revealed that both DHA and EPA affect CASP3 and, consequently induction of apoptosis (Sauer et al., 2005; Xue et al., 2017). Also, the BCL‐2 family proteins are essential in Omega‐3‐induced cell death (Çetin et al., 2021; Lessa et al., 2021; Ma et al., 2021). Our findings are in line with those of previous studies. The results indicated that Omega‐3 inhibited cell viability of colon cancer cell lines more than normal cells. Additionally, it increased the expression of BAX and CASP3, and decreased the expression of the anti‐apoptotic gene of BCL‐2 in cancer cells. These results suggested that Omega‐3 activated caspases and induced apoptosis through the mitochondria‐mediated and death receptor‐mediated apoptotic pathways. Notably, the viability rate of cancer cells was significantly suppressed by the *L. plantarum*. Also, the mRNA level of the BAX gene was upregulated, while BCL‐2 was downregulated in C26 cells after being treated with either suspension of *L. plantarum* or bacteria extract. Therefore, the BAX/BCL‐2 ratio was significantly increased, which may lead to the collapse of mitochondrial membrane potential, resulting in the release of cytochrome c (Cyt) and consequently causing cell apoptosis (Naseri et al., 2015). Additionally, upregulation of CASP3 gene expression in C26 cells was detected in cells being treated with *L. plantarum*, suggesting the activation of mitochondrial apoptotic pathways (Brentnall et al., 2013). Our data are consistent with the previous reports (Jiang et al., 2020; Sentürk et al., 2020; Sun et al., 2021; Yue et al., 2020).

The stability and delivery of bacteria extract to the exact site of action is a crucial factor in cancer treatment. Therefore, it is essential to utilize a suitable method of delivery and stability for effective cancer therapeutics. Microencapsulation of probiotics has been considered a suitable method to improve the survival rate and protection of probiotics in the GI tract. In the present study, the effect of gelatin–gum Arabic microcapsules, gelatin–chitosan microcapsules, and the combined therapy of *L. plantarum* and Omega‐3 without any microencapsulation process on cell viability and apoptosis of cancer cell line in comparison with normal cells were assessed. Our finding showed that the combined therapy of *L. plantarum* and Omega‐3 without any capsulation or containing different capsules had more effect on viability and apoptotic gene expression of cancer cells in comparison with normal cells. Although, these treatments had no more effect on the viability and apoptotic gene expression of cancer cells in comparison with *L. plantarum* or Omega‐3. Nonseparation of *L. plantarum* from Omega‐3 in the compounds may be a probable molecular mechanism for reducing the simultaneous effect of combination therapy and microcapsulated compounds on the apoptosis of the cancer cells. More investigations are required to prove this hypothesis.

## 5 CONCLUSION

The present study showed that Omega‐3 selectively induces cytotoxicity and apoptosis in tumor cells. In addition, the suspension of *L. plantarum* and bacteria extract induced apoptosis in cancer cells more than in normal cells. The combined effect of *L. plantarum* and Omega‐3 did not improve cytotoxicity and apoptosis in colorectal C26 cancer cells compared with monotherapy with *L. plantarum* or Omega‐3.

## FUNDING INFORMATION

This work was supported by the Deputy of Research and Technology of Mazandaran University of Medical Sciences [grant number: 1344].

## CONFLICT OF INTEREST

The authors declare no conflict of interest.

## ETHICAL APPROVAL

The protocol of this study was approved by the Research Ethics Committee of the Mazandaran University of Medical Sciences (IR.MAZUMS.REC.1397.318).

## Data Availability

The data that support the findings of this study are available from the corresponding author upon reasonable request.
